# The Impact of Systemic Inflammation Response Index on the Prognosis of Patients with ST-Segment Elevation Myocardial Infarction Undergoing Percutaneous Coronary Intervention

**DOI:** 10.31083/j.rcm2405153

**Published:** 2023-05-19

**Authors:** Chao Qu, Xiang Li, Hai Gao

**Affiliations:** ^1^Center for Coronary Artery Disease, Division of Cardiology, Beijing Anzhen Hospital, Capital Medical University, 100029 Beijing, China

**Keywords:** systemic inflammation response index (SIRI), ST-segment elevation myocardial infarction (STEMI), 30-day major adverse cardiovascular event (MACE)

## Abstract

**Background:**

Inflammation is essential in cardiovascular disease (CVD) development and progression. A novel inflammatory parameter, the systemic inflammation response index (SIRI), has been proven to predict cancer prognosis strongly. Little is known about the relationship between SIRI and outcomes in patients with ST-segment elevation myocardial infarction (STEMI).

**Methods:**

1312 STEMI patients who underwent percutaneous coronary intervention (PCI) in Beijing Anzhen hospital from January 2019 to December 2021 were analyzed. SIRI was calculated as neutrophils × monocytes/lymphocytes. Our primary outcome was a 30-day major adverse event (MACE), including all-cause mortality, non-fatal myocardial infarction (MI), stroke, incident heart failure (HF), cardiogenic shock, and cardiac arrest.

**Results:**

Patients were stratified into four groups according to quartiles of SIRI: SIRI <1.58 (n = 328), 1.58 ≤ SIRI < 3.28 (n = 328), 3.28 ≤ SIRI < 7.80 (n = 328), SIRI ≥7.80 (n = 328). Higher SIRI was associated with a higher incidence of the 30-day MACE. The rates of 30-day MACE were 6.1%, 8.8%, 12.8%, and 17.1% (*p *< 0.001) for the lowest SIRI quartile to the highest quartile, respectively. This association was consistent in the outcome of HF but no other components. Higher SIRI indicated higher 30-day MACE incidence in most participants except in those with very high inflammatory indicators. Subgroup analysis showed this correlation was consistent in various subgroups (*p* for interaction >0.05).

**Conclusions:**

In patients with STEMI, higher SIRI indicated a higher incidence of 30-day MACE, except for those with very high inflammatory indicators. In most STEMI patients, SIRI might be a trustworthy indicator of short-term prognosis.

## 1. Introduction

Cardiovascular diseases (CVDs) are the leading cause of death, causing an estimated 17.9 million death annually [1]. ST-segment elevation myocardial infarction (STEMI) is one of the most severe conditions of CVDs. Although the overall mortality of STEMI has decreased during the past decades owing to the development of percutaneous coronary intervention (PCI) [2], it remained high at an 8% mortality rate between admission and 1 month after discharge [3]. The most common underlying cause of MI is the rupture or erosion of a coronary atherosclerotic plaque. Inflammation plays an essential role in the initiation and progression of atherosclerosis. Macrophages and T lymphocytes were found highly infiltrated in atherosclerotic lesions and presented when acute plaque rupture occurs. Some inflammatory indicators, such as neutrophile granulocyte count or lymphocyte count, played an important role in predicting the occurrence and prognosis of CVDs [4, 5, 6].

In addition to these traditional indicators, novel inflammatory indicators are also of great value for the prognosis of CVDs. In a previous study, the neutrophile/lymphocyte ratio (NLR) was proven to be associated with mortality and incidence of CVDs [7]. Systemic immune-inflammation index (SII, neutrophil × platelet/lymphocyte) was found to be a potential biomarker for CVD development [8]. Systemic inflammation response index (SIRI) was a novel, noninvasive, easily accessible, and cost-effective index. In previous studies, the prognostic value of SIRI in patients with tumors was widely recognized [9, 10, 11]. Integrating SIRI can predict cervical cancer patients’ survival more accurately and consistently than the standard staging indicator [11]. Whether SIRI is associated with the prognosis of patients with myocardial infarction, especially STEMI, is unknown. Our study aimed to explore the association between SIRI and prognosis in STEMI patients.

## 2. Methods

### 2.1 Study Population

This study is a single-center cohort study among patients diagnosed with STEMI who were treated by PCI in Beijing Anzhen Hospital between January 2019 to December 2021. Patients who met all the following criteria were included in the study: (1) age >18 years old; (2) diagnosed with STEMI according to 2017 ESC guidelines for the management of STEMI [12]; (3) underwent drug-eluting stent (DES) planting after diagnosis. Those who met any of the following criteria were excluded: (1) history of coronary artery bypass grafting; (2) neutrophil data missing; (3) lymphocyte data missing; (4) monocyte data missing; (5) malignant tumor affecting survival; (6) procedure failure of PCI; (7) patients with cardiogenic shock or cardiac arrest before PCI. Finally, 1312 patients were included in the analysis. Informed consent was obtained from every participant in our study.

### 2.2 Data Extraction

The following data were recorded in this study: demographics (age, sex), smoking status, weight, vital signs (heart rate, systolic blood pressure), left ventricular ejection fraction (LVEF), laboratory parameters (white blood cell, neutrophil, lymphocyte, monocyte, hemoglobin, platelet, creatinine, blood nitrogen urea, alanine aminotransferase (ALT), aspartate aminotransferase (AST), sodium, potassium, triglycerides (TG), total cholesterol (TC), low-density lipoprotein cholesterol (LDL-C), high-density lipoprotein cholesterol (HDL-C), high sensitive C reactive protein (hs-CRP)), medication use (aspirin, clopidogrel, ticagrelor, beta-blockers, angiotensin-converting enzyme inhibitor (ACEI), angiotensin receptor blocker (ARB), statins), comorbidities and medical history (hypertension, diabetes, cerebrovascular disease, pneumonia, autoimmune disease), life support equipment (extracorporeal membrane oxygenation (ECOM), Impella, intra-aortic balloon pump (IABP), mechanical ventilation), Killip classification, coronary angiography results (Culprit vessel (left anterior descending artery (LAD); left circumflex artery (LCX); right coronary artery (RCA); left main coronary artery (LM)), number of stents), thrombolysis in myocardial infarction (TIMI) grades. Coronary angiographic data were analyzed and evaluated by optical measurements, and results were recorded and validated by at least two experienced cardiologists.

### 2.3 Grouping and Outcomes

Systemic inflammation response index (SIRI) was defined as neutrophils × monocytes/lymphocytes [9]. All the serological results were obtained from the first blood test report after admission before PCI. According to the SIRI quartiles, all enrolled patients were divided into four groups. The primary outcome was a composite outcome of a 30-day major adverse cardiovascular event (MACE), which included all-cause mortality, non-fatal myocardial infarction (MI), stroke, incident heart failure (HF), cardiogenic shock, and cardiac arrest.

### 2.4 Statistical Analysis

Normally distributed continuous variables were expressed as mean ± standard deviation (SD) and compared between groups using analysis of variance. Skewed data were expressed as median (interquartile range (IQR)) and compared using the Kruskal-Wallis test. Categorical variables were expressed as numbers (percentages) and compared between groups using the Chi-square test.

Multiple logistic regression analysis was used to analyze the relationship between SIRI and 30-day MACE after adjustment for covariates. And the results were expressed as odds ratio (OR) and 95% confidence interval (CI). *p* for trend was calculated. The covariates with *p *< 0.05 in the univariate logistic regression analysis were included in the multivariate logistic regression model. Further, univariate logistic regression analysis was used to explore the relationship between SIRI and 30-day MACE in different inflammatory statuses: status 1: Patients without pneumonia and autoimmune disease; status 2: Patients with WBC (white blood cell) ≥10 × ${\mathrm{10}}^{9}$/L or <4 × ${\mathrm{10}}^{9}$/L and hs-CRP ≥20 mg/L; status 3: Patients with 4 × ${\mathrm{10}}^{9}$/L < WBC ≤ 10 × ${\mathrm{10}}^{9}$/L and hs-CRP <20 mg/L.

Subgroup analysis was used to determine the relationship between SIRI as a continuous variable and 30-day MACE in different subgroups, and *p* for interaction was calculated. The median was considered as the cut-off value in the continuous variable of the subgroup in order to avoid the situation of too few people in a certain subgroup. Univariate logistic analysis was used in subgroup analysis to calculate OR values. In addition, according to the multivariate logistic regression model, we drew the restricted cubic spline (RCS) curve to investigate the association between SIRI as a continuous scale and 30-day MACE. Three knots were chosen for the analysis. Receiver operating characteristic (ROC) analysis was applied to assess the ability of SIRI, NLR, and hs-CRP in predicting the incidence of 30-day MACE. Differences between the area under the ROC curve (AUC) of them were compared using the DeLong test.

All tests were two-sided, and *p *< 0.05 was considered statistically significant. Relevant guidelines and regulations are carried out for all methods. All data analyses were performed by Stata V.15.1 (Statistical Analysis System, Raleigh, NC, USA).

## 3. Results

### 3.1 Subjects and Baseline Characteristics

A total of 1312 STEMI patients who received PCI treatment were included in the study. All the participants were stratified into four groups according to SIRI quartiles: SIRI <1.58 (n = 328), 1.58 ≤ SIRI < 3.28 (n = 328), 3.28 ≤ SIRI < 7.80 (n = 328), SIRI ≥7.80 (n = 328). The characteristics of different groups are summarized in Table 1. Patients in higher SIRI quartiles were more likely to be smokers. Regarding laboratory parameters, patients with a higher SIRI had a higher white blood cell count, neutrophil count, monocyte count, hemoglobin, platelet, creatinine, blood nitrogen urea, ALT, AST, and potassium, whereas lymphocyte count and sodium were lower.

**Table 1.** **Characteristics of study patients by SIRI quartiles**.

Characteristics		Total (n = 1312)	Quartiles of SIRI				*p* value
			Quartile 1 (n = 328)	Quartile 2 (n = 328)	Quartile 3 (n = 328)	Quartile 4 (n = 328)	
			SIRI <1.58	1.58≤ SIRI <3.28	3.28≤ SIRI <7.80	SIRI ≥7.80	
Age (years)		58.4 ± 11.2	57.0 ± 10.9	58.92 ± 10.6	59.1 ± 11.71	58.4 ± 11.4	0.077
Sex, n (%)							0.347
	Male	1059 (80.7)	267 (81.4)	264 (80.5)	273 (83.2)	255 (77.7)	
	Female	253 (19.3)	61 (18.6)	64 (19.5)	55 (16.8)	73 (22.3)	
Smoke, n (%)		421 (32.1)	132 (40.2)	86 (26.2)	109 (33.2)	94 (28.7)	0.001
Weight (kg)		65.1 ± 9.2	65.4 ± 9.0	65.0 ± 9.1	65.6 ± 10.0	64.4 ± 8.8	0.355
Vital signs							
	Heart rate (beats/min)	76.7 ± 17.1	76.7 ± 18.3	77.0 ± 16.8	76.8 ± 15.7	76.2 ± 17.3	0.928
	Systolic blood pressure (mmHg)	135.8 ± 28.3	134.0 ± 29.6	135.6 ± 26.3	138.6 ± 28.8	134.9 ± 28.2	0.186
Ultrasound cardiogram							
	LVEF (%)	52.9 ± 11.8	52.4 ± 12.3	52.6 ± 11.6	53.2 ± 12.0	53.4 ± 11.4	0.674
Laboratory parameters							
	White blood cell (${\mathrm{10}}^{9}$/L)	10.8 ± 5.6	7.4 ± 3.7	8.8 ± 3.4	11.3 ± 4.8	15.8 ± 6.0	<0.001
	Neutrophil (${\mathrm{10}}^{9}$/L)	7.8 ± 4.6	4.2 ± 2.3	6.0 ± 2.4	8.5 ± 3.7	12.5 ± 4.7	<0.001
	Lymphocyte (${\mathrm{10}}^{9}$/L)	1.5 ± 1.3	2.2 ± 1.8	1.5 ± 1.0	1.2 ± 0.8	0.9 ± 0.6	<0.001
	Monocyte (${\mathrm{10}}^{9}$/L)	0.7 ± 0.4	0.5 ± 0.2	0.6 ± 0.3	0.7 ± 0.3	1.0 ± 0.5	<0.001
	Hemoglobin (g/dL)	12.5 ± 2.1	10.4 ± 2.1	12.1 ± 1.5	13.3 ± 1.2	14.1 ± 1.2	<0.001
	Platelet (${\mathrm{10}}^{9}$/L)	220.6 ± 102.2	207.5 ± 87.7	217.2 ± 91.4	224.1 ± 103.3	233.6 ± 121.5	0.009
	Creatinine (mg/dL)	1.0 [0.8, 1.5]	0.9 [0.7, 1.2]	1.0 [0.8, 1.6]	1.0 [0.8, 1.5]	1.2 [0.8, 1.9]	<0.001
	Blood nitrogen urea (mg/dL)	26.7 ± 22.2	21.6 ± 18.3	25.7 ± 21.0	27.2 ± 21.4	32.3 ± 26.2	<0.001
	ALT (U/L)	25.5 [16.4, 40.9]	25.5 [17.3, 38.2]	23.6 [16.4, 36.4]	24.6 [16.4, 37.7]	27.7 [16.4, 53.6]	0.009
	AST (U/L)	25.5 [18.2, 46.4]	24.6 [16.4, 40.0]	23.6 [17.2, 40.0]	25.2 [18.2, 47.3]	32.7 [20.0, 66.1]	<0.001
	Sodium (mmol/L)	137.4 ± 5.6	138.4 ± 4.2	138.0 ± 5.9	136.8 ± 5.5	136.4 ± 6.3	<0.001
	Potassium (mmol/L)	4.2 ± 0.8	4.1 ± 0.7	4.2 ± 0.7	4.2 ± 0.8	4.3 ± 0.8	0.003
	TG (mg/dL)	110.0 [78.0, 157.0]	114.0 [81.0, 160.0]	113.5 [82.0, 162.3]	108.0 [73.5, 154.3]	105.5 [72.6, 152.3]	0.224
	TC (mg/dL)	153.1 ± 45.7	156.3 ± 46.5	152.5 ± 45.5	149.8 ± 45.2	153.7 ± 45.4	0.335
	LDL-C (mg/dL)	81.6 ± 35.7	85.5 ± 36.5	80.6 ± 35.1	78.2 ± 34.9	82.2 ± 35.9	0.062
	HDL-C (mg/dL)	41.2 ± 16.3	40.5 ± 15.7	40.1 ± 16.2	42.0 ± 15.8	42.0 ± 17.3	0.318
	hs-CRP (mg/L)	3.7 [1.3, 11.6]	2.8 [0.8, 8.3]	3.1 [1.0, 11.8]	4.0 [1.8, 12.5]	6.3 [2.1, 16.1]	<0.001
Medication use, n (%)							
	Aspirin	1303 (99.3)	326 (99.4)	326 (99.4)	327 (99.7)	324 (98.8)	0.547
	Clopidogrel	1016 (77.4)	255 (77.7)	252 (76.8)	258 (78.7)	251 (76.5)	0.914
	Ticagrelor	206 (15.7)	54 (16.5)	49 (14.9)	46 (14.0)	57 (17.4)	0.641
	Beta-blockers	902 (68.8)	222 (67.7)	231 (70.4)	224 (68.3)	225 (68.6)	0.888
	ACEI	638 (48.6)	166 (50.6)	150 (45.7)	173 (52.7)	149 (45.4)	0.159
	ARB	134 (10.2)	34 (10.4)	43 (13.1)	24 (7.3)	33 (10.1)	0.111
	Statins	1290 (98.3)	324 (98.8)	321 (97.9)	319 (97.3)	326 (99.4)	0.147
Comorbidities and medical history, n (%)							
	Hypertension	555 (42.3)	127 (38.7)	140 (42.7)	147 (44.8)	141 (43.0)	0.447
	Diabetes	564 (43.0)	141 (43.0)	150 (45.7)	127 (38.7)	146 (44.5)	0.289
	Cerebrovascular disease	11 (0.8)	3 (0.9)	2 (0.6)	3 (0.9)	3 (0.9)	0.965
	Pneumonia	29 (1.21)	1 (0.3)	2 (0.6)	13 (4.0)	13 (4.0)	<0.001
	Autoimmune disease	14 (1.1)	2 (0.6)	2 (0.6)	4 (1.2)	6 (1.8)	0.365
Life support equipment, n (%)		28 (2.1)	9 (2.7)	6 (1.8)	6 (1.8)	7 (2.1)	0.831
Killip classification, n (%)							0.465
	I	1174 (89.5)	294 (89.6)	294 (89.6)	302 (92.1)	284 (86.6)	
	II	81 (6.2)	18 (5.5)	23 (7.0)	16 (4.9)	24 (7.3)	
	III	32 (2.4)	9 (2.7)	5 (1.5)	5 (1.5)	13 (4.0)	
	IV	25 (1.9)	7 (2.1)	6 (1.8)	5 (1.5)	7 (2.1)	
Culprit vessel, n (%)							
	LAD	693 (52.8)	178 (54.3)	170 (51.8)	181 (55.2)	164 (50.0)	0.535
	LCX	158 (12.0)	36 (11.0)	35 (10.7)	41 (12.5)	46 (14.0)	0.529
	RCA	441 (33.6)	108 (32.9)	122 (37.2)	102 (31.1)	109 (33.2)	0.406
	LM	20 (1.5)	6 (1.8)	1 (0.3)	4 (1.2)	9 (2.7)	0.075
Stent numbers, n (%)							0.517
	1	1232 (93.9)	313 (95.4)	309 (94.2)	306 (93.3)	304 (92.7)	
	2	75 (5.7)	13 (4.0)	18 (5.5)	21 (6.4)	23 (7.0)	
	3	4 (0.3)	2 (0.6)	1 (0.3)	1 (0.3)	0 (0.0)	
	4	1 (0.1)	0 (0.0)	0 (0.0)	0 (0.0)	1 (0.3)	
TIMI grades, n (%)							<0.001
	II	155 (11.8)	31 (9.5)	27 (8.2)	38 (11.6)	59 (18.0)	
	III	1157 (88.2)	297 (90.6)	301 (91.8)	290 (88.4)	269 (82.0)	

Continuous variables were presented as mean ± SD or median (IQR). Categorical variables were presented as numbers (percentages). Skewed data were expressed as median (interquartile range (IQR)). Life support equipment: Extracorporeal membrane oxygenation (ECOM), Impella, intra-aortic balloon pump (IABP), mechanical ventilation. Abbreviation: SIRI, systemic inflammation response index; LVEF, left ventricular ejection fraction; ALT, alanine aminotransferase; AST, aspartate aminotransferase; LDL-C, low-density lipoprotein cholesterol; HDL-C, high-density lipoprotein cholesterol; TG, triglyceride; TC, total cholesterol; hs-CRP, high sensitive C reactive protein; ACEI, angiotensin-converting enzyme inhibitor; ARB, angiotensin receptor blocker; LAD, left anterior descending artery; LCX, left circumflex artery; RCA, right coronary artery; LM, left main coronary artery; TIMI, thrombolysis in myocardial infarction.

### 3.2 Association between SIRI and 30-Day MACE

As shown in Table 2, the incidence of 30-day MACE was 11.2%, and a higher SIRI was related to a significant increase in the rate of 30-day MACE (quartile 4 vs. quartile 1: 17.1% vs. 6.1%, *p *< 0.001). The rate of incident HF was 4.2%, and we found a significant trend toward increased risk of incident HF with increasing SIRI (quartile 4 vs. quartile 1: 6.4% vs. 1.8%, *p* = 0.027). The rates of all-cause death, non-fatal MI, stroke, cardiogenic shock, and cardiac arrest were 4.4%, 1.1%, 0.8%, 3.1%, and 2.3%, respectively. However, we failed to demonstrate that an elevated SIRI quartile was significantly associated with an increased rate of all-cause death (quartile 4 vs. quartile 1: 5.5% vs. 3.7%, *p* = 0.583), non-fatal MI (quartile 4 vs. quartile 1: 2.1% vs. 0.3%, *p* = 0.105), stroke (quartile 4 vs. quartile 1: 1.2% vs. 0.3%, *p* = 0.305), cardiogenic shock (quartile 4 vs. quartile 1: 5.2% vs. 2.1%, *p* = 0.097) and cardiac arrest (quartile 4 vs. quartile 1: 2.7% vs. 1.8%, *p* = 0.746).

**Table 2.** **Clinical outcomes of the study patients by SIRI quartiles**.

Outcomes	Total (n = 1312)	Quartiles of SIRI				*p* value
		Quartile 1 (n = 328)	Quartile 2 (n = 328)	Quartile 3 (n = 328)	Quartile 4 (n = 328)	
		SIRI <1.58	1.58≤ SIRI <3.28	3.28≤ SIRI <7.80	SIRI ≥7.80	
30-day MACE, n (%)	147 (11.2)	20 (6.1)	29 (8.8)	42 (12.8)	56 (17.1)	<0.001
All-cause death	58 (4.4)	12 (3.7)	16 (4.9)	12 (3.7)	18 (5.5)	0.583
Non‑fatal MI	15 (1.1)	1 (0.3)	2 (0.6)	5 (1.5)	7 (2.1)	0.105
Stoke	10 (0.8)	1 (0.3)	1 (0.3)	4 (1.2)	4 (1.2)	0.305
Incident heart failure	55 (4.2)	6 (1.8)	12 (3.7)	16 (4.9)	21 (6.4)	0.027
Cardiogenic shock	41 (3.1)	7 (2.1)	8 (2.4)	9 (2.7)	17 (5.2)	0.097
Cardiac arrest	30 (2.3)	6 (1.8)	9 (2.7)	6 (1.8)	9 (2.7)	0.746

Categorical variables were presented as numbers (percentages). *p* values were calculated using Chi-square test to compare differences in outcomes between different SIRI quartiles. Abbreviation: SIRI, systemic inflammation response index; MACE, major adverse cardiovascular event; MI, myocardial infarction.

In multiple logistic regression analysis, adjusted for confounding variables, a positive correlation was noted between SIRI and 30-day MACE (quartile 4 vs. quartile 1: OR, 95% CI: 3.30, 1.55–7.03, *p *= 0.002, *p* for trend <0.001). In addition, we found that diabetes (OR, 95% CI: 1.53, 1.04–2.25, *p* = 0.029), higher LDL-C (OR, 95% CI: 1.01, 1.00–1.01, *p* = 0.002), and Killip class (OR, 95% CI: 1.48, 1.15–1.91, *p* = 0.003) were significantly associated with an increased risk of 30-day MACE respectively. While women (female vs. male: OR, 95% CI: 0.56, 0.35–0.90, *p* = 0.016), higher systolic blood pressure (OR, 95% CI: 0.99, 0.98–1.00, *p* = 0.002) and LVEF (OR, 95% CI: 0.94, 0.93–0.96, *p *< 0.001) were significantly related to the reduction in the risk of 30-day MACE respectively (Table 3).

**Table 3.** **The association between SIRI and incidence of 30-day MACE in logistic analysis model**.

Variables	OR (95% CI)	*p* value	*p* for trend
SIRI			<0.001
Quartile 1	Reference		
Quartile 2	1.61 (0.83–3.15)	0.161	
Quartile 3	2.82 (1.39–5.72)	0.004	
Quartile 4	3.30 (1.55–7.03)	0.002	
Age	1.01 (0.99–1.03)	0.317	
Sex (Female)	0.56 (0.35–0.90)	0.016	
Systolic blood pressure	0.99 (0.98–1.00)	0.002	
LVEF	0.94 (0.93–0.96)	<0.001	
Hemoglobin	1.06 (0.93–1.21)	0.351	
Potassium	0.79 (0.61–1.02)	0.072	
LDL-C	1.01 (1.00–1.01)	0.002	
HDL-C	1.00 (0.98–1.01)	0.577	
TG	1.00 (1.00–1.00)	0.909	
Smoke	1.50 (0.97–2.31)	0.068	
Diabetes	1.53 (1.04–2.25)	0.029	
Killip classification	1.48 (1.15–1.91)	0.003	
hs-CRP	1.00 (0.99–1.01)	0.666	
TIMI grades	1.02 (0.57–1.81)	0.959	

Model was derived from multivariate logistic regression analysis. Abbreviation: SIRI, systemic inflammation response index; MACE, major adverse cardiovascular event; LVEF, left ventricular ejection fraction; LDL-C, low-density lipoprotein cholesterol; HDL-C, high-density lipoprotein cholesterol; TG, triglyceride; hs-CRP, high sensitive C reactive protein; TIMI, thrombolysis in myocardial infarction; OR, odd ratio; CI, confidence interval.

**Fig. 1.**
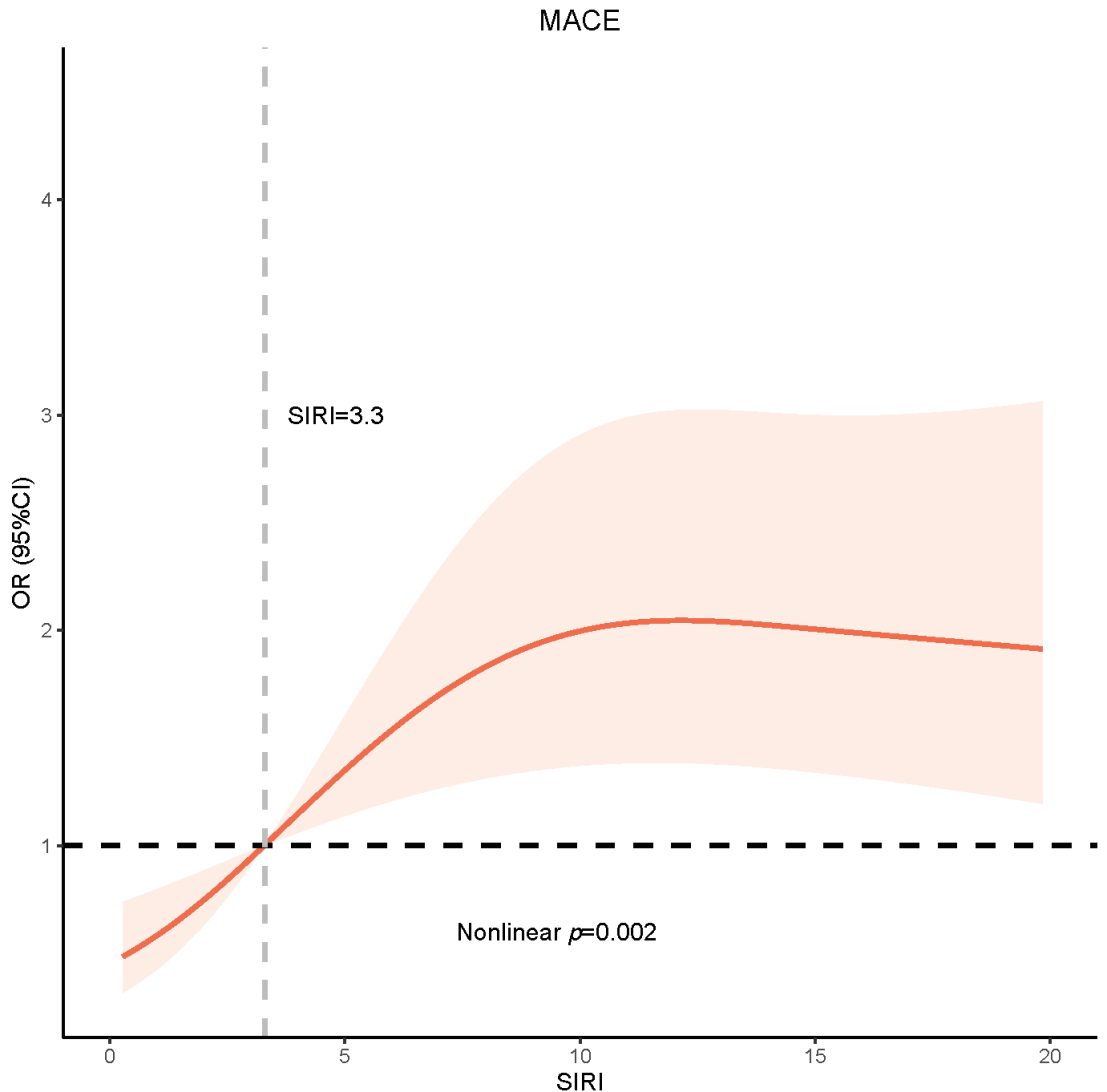
**RCS model showing the association between the SIRI and MACE**. Abbreviation: SIRI, systemic inflammation response index; MACE, major adverse cardiovascular event; RCS, restricted cubic spline.

In Fig. 1, we used the RCS model to analyze the nonlinear relationship between 30-day MACE and SIRI as a continuous variable (Non-linear *p* = 0.002). The results showed a positive relationship between SIRI and the risk of 30-day MACE after adjustment for potential confounders in the model.

### 3.3 Association between SIRI and 30-Day MACE in Different Inflammatory Status

The association between SIRI and the incidence of 30-day MACE in a different state of inflammation is shown in Table 4. We found that higher quartiles of SIRI were significantly associated with an increased risk of 30-day MACE in all statuses except status 2, suggesting that SIRI was a significant prognosis marker of 30-day MACE in mild or non-inflammatory status.

**Table 4.** **The association between SIRI and incidence of 30-day MACE in different status of inflammation**.

Classification	N	Quartile 1	Quartile 2	Quartile 3	Quartile 4	*p* for trend
Status 1	1271	Reference	1.58 (0.87–2.89)	2.44 (1.39–4.31)	3.50 (2.02–6.05)	<0.001
Status 2	144	Reference	4.92 (0.41–59.11)	2.13 (0.18–24.76)	4.57 (0.55–38.23)	0.164
Status 3	576	Reference	1.47 (0.67–3.22)	3.11 (1.47–6.60)	3.48 (1.13–10.70)	0.001

Binary logistic regression analysis was used and results were presented as OR (odds ratio) and 95% CI (confidence interval). All patients were divided into 3 subgroups for analysis based on the inflammatory status: status 1: Patients without pneumonia and autoimmune disease; status 2: Patients with WBC (white blood cell) ≥10 × ${\mathrm{10}}^{9}$/L or <4 × ${\mathrm{10}}^{9}$/L and hs-CRP (high sensitive C reactive protein) ≥20 mg/L; status 3: Patients with 4 × ${\mathrm{10}}^{9}$/L < WBC ≤ 10 × ${\mathrm{10}}^{9}$/L and hs-CRP <20 mg/L.

### 3.4 The Area under the ROC Curve (AUC) of Different Indicators

**Fig. 2.**
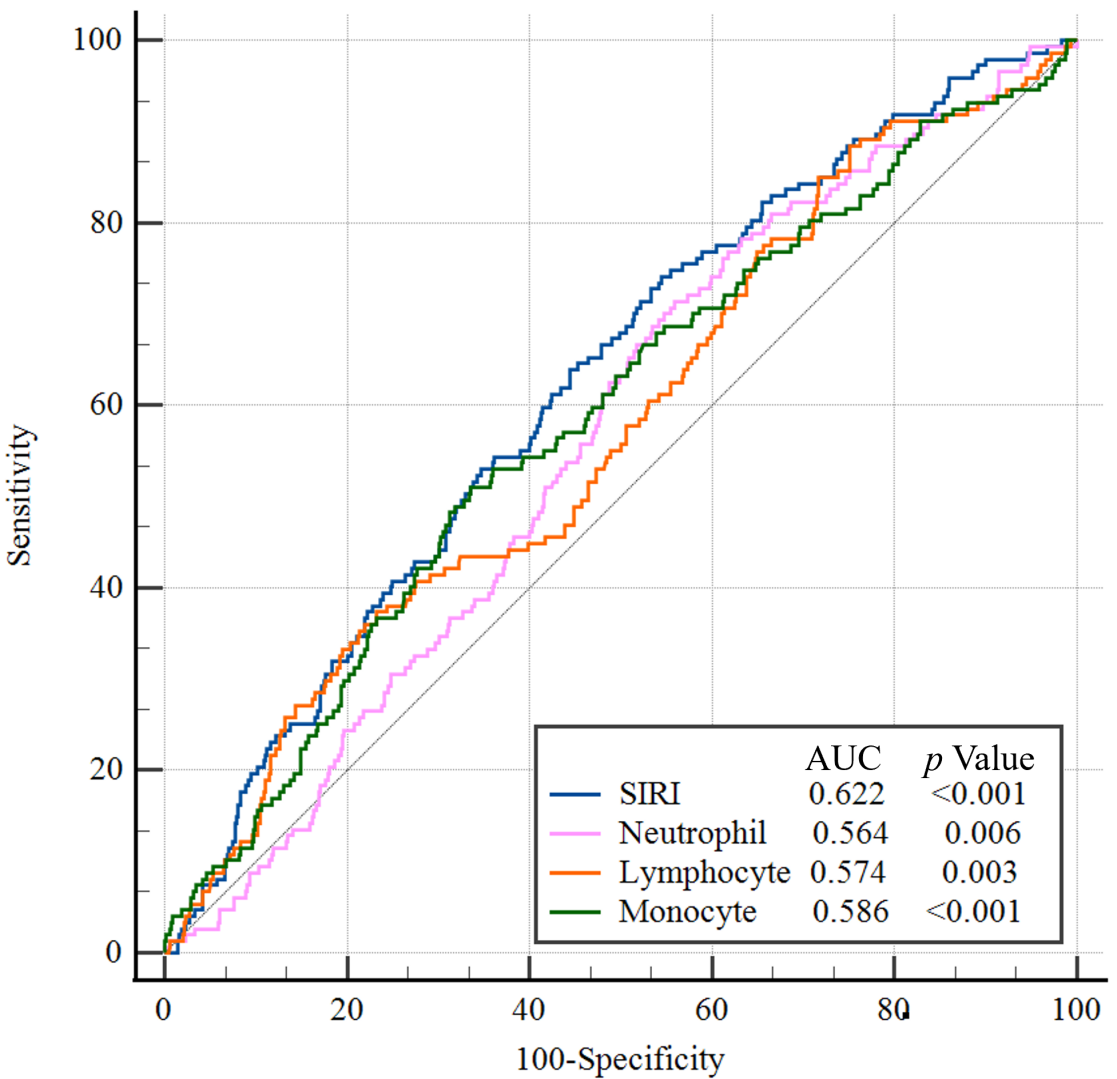
**ROC curves for the prediction of 30-day MACE of SIRI, NLR and hs-CRP**. Abbreviation: ROC, receiver operating characteristic; MACE, major adverse cardiovascular event; SIRI, systemic inflammation response index; NLR, neutrophil to lymphocyte ratio; hs-CRP, high sensitive C reactive protein.

The ability to predict the 30-day MACE of SIRI, NLR and hs-CRP was presented in Fig. 2. The AUCs of SIRI for 30-day MACE was 0.622, which was larger than the AUC of the NLR (De-long test, *p *= 0.046) and hs-CRP (De-long test, *p* = 0.015) respectively, suggesting that SIRI had the better predictive accuracy of adverse outcomes in patients with STEMI.

### 3.5 Subgroup Analysis

**Fig. 3.**
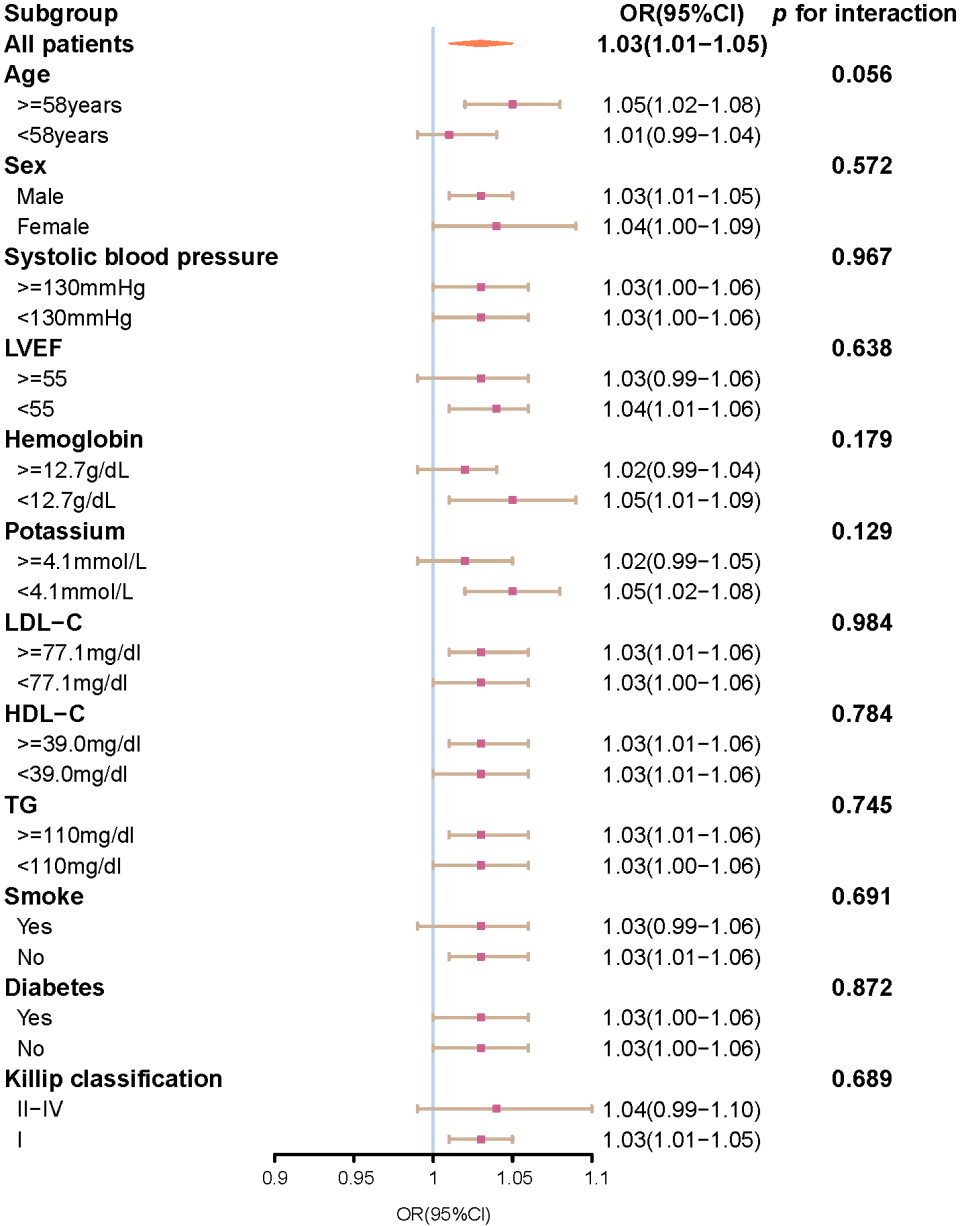
**Subgroup analysis of the association between 30-day MACE and SIRI as a continuous variable**. Abbreviation: OR, odds ratio; CI, confidence interval; SIRI, systemic inflammation response index; MACE, major adverse cardiovascular event.

In subgroup analysis, the numerical SIRI was positively associated with a higher risk of 30-day MACE in all subgroups. Moreover, no significant interactions were observed in all subgroup analyses (Fig. 3).

## 4. Discussion

Our study was focused on the correlation between SIRI and short-term prognosis in STEMI patients undergoing PCI. Patients with higher SIRI were in more severe inflammatory conditions. Higher SIRI was associated with a higher incidence of 30-day MACE except in severe inflammatory conditions. After adjusting for main confounders, SIRI was still associated with 30-day MACE, and the association was homogeneous among different subgroups.

Previous studies have demonstrated that inflammation has a crucial role in the development of atherosclerosis. The infiltration of low-density lipoprotein (LDL) in the arterial wall initiated the inflammatory response [13]. Plasma-derived lipoproteins infiltrated tissues and were modified by macrophages. These lipid-filled foam cells triggered atherosclerotic lesion formation. The progression of the lesion is then maintained by the insufficient efferocytotic removal of foam cells and apoptotic cells [14]. Also, higher neutrophil counts in rupture-prone lesions were shown in the human thin fibrous cap atheroma specimens, indicating a contribution of neutrophils to plaque destabilization [15]. Lymphocytes have also been shown to involving in the acceleration of atherosclerosis [16].

Higher inflammatory indexes were associated with a worse prognosis in patients with acute myocardial infarction. Higher total white blood cells, neutrophils, and monocyte were associated with higher mortality in acute myocardial infarction (AMI). Among all the subtypes, neutrophils correlated best with mortality [17]. Recently, novel indicators also showed prognostic value in CVDs. An observational study showed neutrophil to HDL-C ratio (NHR) could predict long-term outcomes better than traditional indicators in AMI [18]. Another observational study showed neutrophil to lymphocyte ratio was an independent predictor of both in-hospital and long-term adverse outcomes among STEMI patients undergoing PCI [19]. SIRI, as a new inflammatory index, including neutrophils, monocytes, and lymphocytes, had been well recognized in the progress prediction in cancer. In patients with pancreatic adenocarcinomas who receive chemotherapy, those with higher SIRI had a shorter survival time than those with lower SIRI [9]. Poor prognosis was also correlated with higher SIRI in cervical cancer and esophageal squamous cell carcinoma [11, 20].

A few studies have explored the relationship between SIRI and outcomes in patients with CVD. Analysis from a large, prospective, population-based study, the Kailuan study, demonstrated that, in general people, higher SIRI was associated with higher AMI incidence and all-cause mortality, and this association remained even after adjusting reactive protein (CRP) [21]. Zhang *et al*. [22] found that higher SIRI was associated with worse outcomes in stroke patients, including in-hospital mortality, 30-day, 90-day, and one-year mortality, and stroke severity. Han *et al*. [15] demonstrated that SIRI was an independent predictor of MACE and provided incremental prognostic information in patients with acute coronary syndrome (ACS) undergoing PCI. Our study is the first to explore the correlation between SIRI and short-term outcomes in STEMI patients undergoing PCI. In STEMI patients, more plaque rupture and thin cap fibroatheroma were identified compared to NSTEMI/UA or stable coronary artery disease (CAD) lesions. Also, STEMI lesions were identified with a smaller minimum lumen cross-sectional area but a larger plaque burden and positive remodeling [23]. Observational studies found a higher value of highly sensitive, reactive protein (hs-CRP), WBC, ferritin, and IL-6 in STEMI compared to NSTEMI, indicating a differential inflammatory pattern in these two kinds of patients [24]. Dziedzic *et al*. [25] investigated the association between SIRI and the severity of CVD and found that SIRI was significantly higher in ACS than in stable CAD. The highest SIRI was observed in patients with three-vessel CAD.

Our study on STEMI patients undergoing PCI found that higher SIRI was significantly associated with higher 30-day MACE in STEMI patients. Also, the RCS model showed a positive relationship between SIRI and the risk of 30-day MACE. This association was consistent in the outcome of HF but not in other components of MACE, including non-fatal MI, stroke, cardiogenic shock, and cardiac arrest. Circulating monocytes penetrated the myocardium quickly after myocardial infarction and took part in inflammatory and healing processes, which impacted left ventricular remodeling [26]. Inflammation was an important reason for myocardial disorder and played a crucial part in the development of HF [27]. A case-control study that included 385 HF patients showed that hs-CRP, lymphocyte-to-monocyte ratio, and monocyte-to-high-density-lipoprotein ratio were considered independent predictors of the incidence of HF [28], which was consistent with our study. As an easily accessible and cheap parameter, SIRI might be a valuable marker of adverse events in patients with AMI.

Different inflammatory states may also affect SIRI’s predictive value for AMI patients. In our study, higher SIRI was associated with 30-day MACE in mild or no inflammatory status. In those with WBC ≥10 × ${\mathrm{10}}^{9}$/L or <4 × ${\mathrm{10}}^{9}$/L and hs-CRP ≥20 mg/L, this association did not exit. This might be because, in high inflammation status, indicators of inflammation reflect the degree of disease activity like underlying infection or autoimmune disease more than the severity of AMI. As a result, many studies excluded those with infection when exploring the correlation between inflammatory factors and CVD outcomes [29, 30]. The previous study focused on trajectory of CRP after AMI showed a peak of 12.10 mg/L during hospitalization [29]. In our study, patients in status two had hs-CRP over 20 mg/L, and this very high hs-CRP might not only be attributed to AMI.

### Limitations

First, our study was a retrospective observational study with patients from one center. Some selection bias might be inevitable. Second, factors influencing AMI outcomes were various, and variables in our study might have been inadequately collected. Finally, we only had the short-term outcome of 30-day MACE in our study; the relationship between long-term outcomes and SIRI should be further explored.

## 5. Conclusions

Higher SIRI was associated with a higher incidence of 30-day MACE in patients with STEMI. SIRI might be a significant predictor of short-term outcomes in STEMI patients.

## Data Availability

The datasets used and analyzed during the current study are available from the corresponding author on reasonable request.
